# Identification of candidate genes controlling chilling tolerance of rice in the cold region at the booting stage by BSA-Seq and RNA-Seq

**DOI:** 10.1098/rsos.201081

**Published:** 2020-11-18

**Authors:** Zhenhua Guo, Lijun Cai, Zhiqiang Chen, Ruiying Wang, Lanming Zhang, Shiwu Guan, Shuhua Zhang, Wendong Ma, Chuanxue Liu, Guojun Pan

**Affiliations:** 1 Rice Research Institute of Heilongjiang Academy of Agricultural Sciences, Jiamusi 154026, People's Republic of China; 2 National Engineering Research Center of Plant Space Breeding, South China Agricultural University, Guangzhou 510642, People's Republic of China; 3 Jiamusi Branch of Heilongjiang Academy of Agricultural Sciences, Jiamusi 154007, People's Republic of China

**Keywords:** *Oryza sativa*, chilling tolerance, candidate gene, BSA-Seq, RNA-Seq

## Abstract

Rice is sensitive to low temperatures, specifically at the booting stage. Chilling tolerance of rice is a quantitative trait loci that is governed by multiple genes, and thus, its precise identification through the conventional methods is an arduous task. In this study, we investigated the candidate genes related to chilling tolerance at the booting stage of rice. The F2 population was derived from Longjing25 (chilling-tolerant) and Longjing11 (chilling-sensitive) cross. Two bulked segregant analysis pools were constructed. A 0.82 Mb region containing 98 annotated genes on chromosomes 6 and 9 was recognized as the candidate region associated with chilling tolerance of rice at the booting stage. Transcriptomic analysis of Longjing25 and Longjing11 revealed 50 differentially expressed genes (DEGs) on the candidate intervals. KEGG pathway enrichment analysis of DEGs was performed. Nine pathways were found to be enriched, which contained 10 DEGs. A total of four genes had different expression patterns or levels between Longjing25 and Longjing11. Four out of the 10 DEGs were considered as potential candidate genes for chilling tolerance. This study will assist in the cloning of the candidate genes responsible for chilling tolerance and molecular breeding of rice for the development of chilling-tolerant rice varieties.

## 1. Introduction

Rice (*Oryza sativa* L.) is one of the most important crops and serves as a staple food for a large proportion of the population worldwide [1]. Due to its evolutionary pathway through the tropical and subtropical regions, rice is sensitive to low temperatures [2]. Chilling stress occurs in the *Indica* and *Japonica* rice when the temperature falls below 18°C and 15°C, respectively [3]. It mainly occurs at high-altitude areas of tropical and subtropical zones and high-latitude areas of subfrigid zones. Currently, chilling stress is a global concern as far as the cultivation of rice at a lower temperature is concerned. The low temperature hampers around 15 million hm^2^ of rice cultivation, and about 24 countries, especially Japan, Korea and northeastern China, are impacted by the chilling stress in rice [4].

The sensitivity of rice to low temperature makes it more susceptible to chilling stress throughout its developmental phases, particularly during the booting stage. It leads to pollen sterility and thus, the loss in yield. Rice booting stage can be categorized into two stages: the young microspore (YM) and the early binucleate (EB) stage. The YM stage is highly sensitive to low temperatures and more vulnerable to pollen fertility under chilling stress, as tetrads are converted to early uninucleate cells during this stage [5–7].

Spikelet fertility (percentage of fertile grains related to florets) is a commonly used method to evaluate the chilling tolerance of rice during the booting stage by using indoors (greenhouse cultivation) or outdoors (cool water irrigation) methods [8,9]. The cool water irrigation method has been widely used in the evaluation and selection of chilling-tolerant rice varieties for over 30 years due to its high reliability [4,10,11].

The booting stage is the most critical stage of the rice growth phase, and so chilling tolerance at the booting stage has drawn significant attention from the scientific community over time. The modification and regulation of chilling stress in rice at the booting stage is governed by complex regulatory mechanisms and multiple metabolic pathways. During the booting stage, apart from the changing spikelet fertility, the metabolites, such as amino acids, heat-shock proteins, carbohydrates, reactive oxygen species (ROS), antioxidants, Ca^2+^, plant hormones (abscisic acid and gibberellins) and so on, are also involved in response to chilling stress [6,12–15]. *OSINV4* [6] and *Osg1* [16] encode cell-wall invertase and β-1,3-glucanase, respectively, suppression of which could lead to the obstruction of starch formation in pollen grains and pollen sterility. Additionally, other transcription factors and proteins, such as ABRE binding protein [17], bZIP transcription factors [18], calmodulin-like gene *OsMSR2* [19] and so on, are also involved in chilling stress response.

A plethora of studies suggests that chilling tolerance is a heritable trait in rice, which can be stably inherited in subsequent generations [20]. In the last decade, studies on the chilling tolerance of rice during the booting stage were primarily focused on the quantitative trait loci (QTL) location of chilling resistance. To locate the QTL related to chilling tolerance, Andaya & Mackill constructed a genetic map (1276.8 cM length and 175 SSR markers) by employing 191 recombinant inbred lines (RILs) acquired from M-202 (temperate *Japonica* variety) and IR50 (tropical *Indica* variety) cross. The two QTLs, *qCTB2* and *qCTB3*, contribute most to the variations [21]. Till now, more than 59 QTLs were correlated to the chilling tolerance in rice at the booting stage. They explained 0.8–37.8% of the phenotypic variation, which could not be detected in different environments or plant types stably. It suggests that the chilling tolerance of rice during the booting stage is a complex quantitative trait, which is regulated by multiple genes [22].

Currently, only three QTLs, *Ctb1*, *CTB4a* and *qPSR10*, have been cloned and functionally characterized [23–25]. *Ctb1* is the first gene to be identified that is related to chilling tolerance at the booting stage of rice. The F-box protein encoded by *Ctb1* interacts with the subunit SKP1 of the E3 ubiquitin ligase complex [23]. *CTB4a* encodes a conserved leucine-rich repeat receptor-like kinase, which interacts with AtpB, a subunit of ATP synthase. Overexpression of AtpB enhances the ATP synthase activity, seed setting rates (SSRs) and yield during chilling stress [24]. *qPSR10*, containing an SNP, SNP2G, was identified through genome-wide association study (GWAS) technique, which is responsible for conferring chilling tolerance to rice both on the seeding and booting stages [25]. In addition, since the traditional method of QTL location needs advanced generation, which is time-consuming and arduous construction, some other breeding strategies are developed to improve the efficiency of QTL location.

Michelmore *et al*. [26] first described the bulked segregant analysis (BSA). It is a rapid and efficient strategy to identify molecular markers that are closely linked to the target QTLs or genes [26]. The BSA method led to the construction of two bulks of individuals with extreme phenotypes that can rapidly identify the regions closely linked to the target traits. Besides, it averts the time-consuming task of construction of the population for QTL mapping. Previous studies have successfully used the BSA method in rice [27,28], rapeseed [29–31], barley [32,33] and so on. The BSA-Seq strategy is an amalgamation of the BSA method and next-generation sequencing (NGS) technique. It has been widely used in QTL mapping and for the precise identification of target genes, based on the single nucleotide polymorphism (SNP) generated by whole-genome sequencing of two groups of bulked samples [34,35]. This strategy has been used in different types of plants, such as rice [36], cotton [37], cucumber [38–40], soya bean [41] and maize [42].

Heilongjiang province, located in the northernmost part of China, is a major producer of rice in China. Chilling stress, especially during the booting stage, impacts the total rice production of Heilongjiang province [43]. Therefore, chilling tolerance breeding in rice is an important strategy to improve rice production in Heilongjiang province. Due to the genetic complexity of chilling tolerance in rice at the booting stage and limitation of the traditional QTL mapping method, in the current study, we employed RNA-sequencing (RNA-Seq) and BSA-sequencing (BSA-Seq). These sequencing technologies were integrated to identify the genes controlling the chilling tolerance at the booting stage in the F_2_ segregating population obtained from a cross between a Longjing25 (LJ25, chilling-tolerant variety) and Longjng11 (LJ11, chilling-sensitive variety). In the current investigation, we identified the differentially expressed genes (DEGs) related to chilling stress and the regions of genes associated with the chilling tolerance at the booting stage. We also validated the candidate genes, which can be cloned and functionally analysed for the development of chilling-tolerant rice varieties.

## 2. Results

### 2.1. Effect of chilling treatment on morphological and physiological characteristics of LJ25 and LJ11 indoors

**Figure 1.**
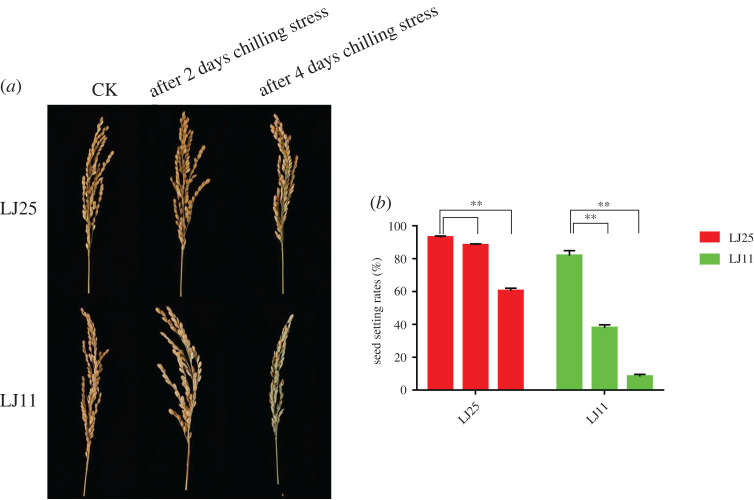
LJ25 and LJ11 phenotypes under chilling stress. (*a*) Comparison of LJ25 and LJ11 panicles: control (day 0) versus chilling stress treatment at 12°C for 2 and 4 days. (*b*) Comparison of the LJ25 and LJ11 SSRs: control versus chilling stress treatment. CK represents the panicles in the control group without chilling stress treatment. Each three columns of LJ25 and LJ11 from left to right in the histogram represent the SSRs on 0, 2 and 4 days of chilling stress treatment, respectively. Asterisks represent statistically significant differences (**p < 0.01) between the control (0 day) and treatment groups (2 and 4 days) in LJ25 and LJ11.

In the current study, we assessed the SSRs of the two cultivars, i.e. LJ25 and LJ11, on days 2 and 4 after chilling stress (12°C), in indoor greenhouse cultivation. In LJ25, 2 days after chilling stress, SSRs did not decrease significantly. However, 4 days after chilling stress, few sterile spikelets were present on the panicles of LJ25 with a significant decrease in SSR (SSRs: 93.00%, 89.27% and 60.35% on days 0, 2 and 4, respectively). In LJ11, 2 days after chilling stress, more than half of the spikelets, and 4 days after chilling stress, almost all spikelets were sterile on the panicles due to the chilling stress. The chilling stress led to a significant decrease in SSRs of LJ11 (SSRs: 90.05%, 37.85% and 8.3% on days 0, 2 and 4, respectively) (figure 1).

### 2.2. Phenotypic variation of SSRs of F2 population and segregating pools construction

**Figure 2.**
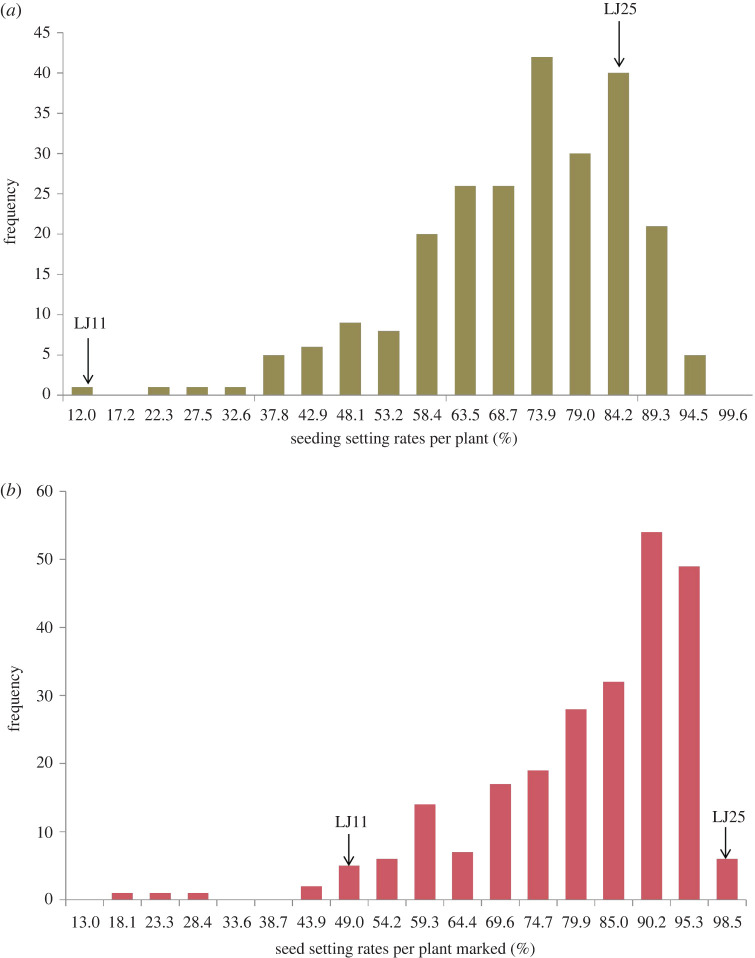
Frequency distribution of SSRPP and SSRPPM of the F2 population derived from the cross between LJ25 and LJ11: (*a*) SSRPP and (*b*) SSRPPM. Arrows represent the mean phenotypic values of both parental lines.

**Figure 3.**
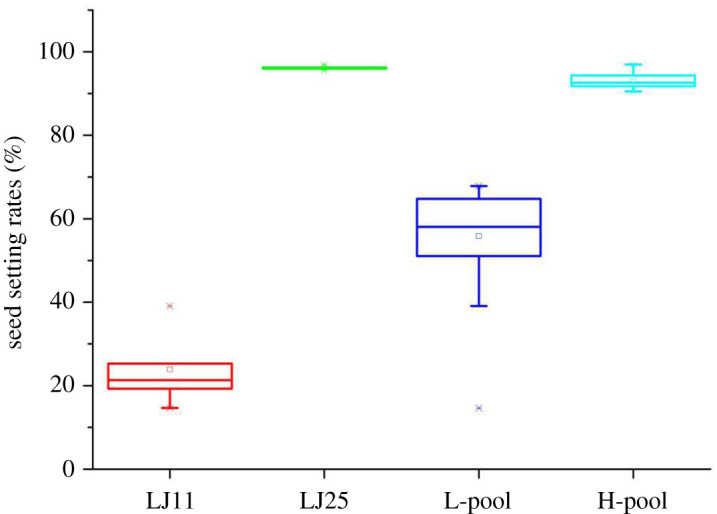
Boxplots represent SSRPPM of two BSA extremity pools and parental lines. L-pool/H-pool showed low/high SSRPPM extremities. The band inside the boxes indicates the median.

The seed setting rates per plant (SSRPP) and seed setting rates per plant marked (SSRPPM) of each plant in the F2 population were found to be in the range of 10.67–93.14% and 14.43–96.26%, respectively. In LJ11 and LJ25, it was found to be in the range of 10.67–39% and 83.44–96.65%, respectively. As shown in figure 2, the SSRPP and the SSRPPM were uniformly dispersed as a single peak. Besides, extensive variation with continuous distributions was observed, which indicated that both were quantitative traits controlled by multiple genes. As SSRPP and the SSRPPM displayed similar trends, the SSRPPM value was set as the criteria to segregate the pool's construction. Fifty individuals with high chilling tolerance (SSRPPM: 90.30–96.26%) and 50 individuals with high chilling sensitivity (SSRPPM: 14.65–68.13%) were selected to prepare the H-pool and L-pool, respectively (figure 3; electronic supplementary material, table S1). The average of the SSRPPM was highest in LJ25 (96.95%), followed by the H-pool (92.88%) and L-pool (56.45%); it was lowest in LJ11 (39.09%) (figure 3).

### 2.3. BSA-Seq data analysis and reads assembly

A total of 224.11 million raw reads were acquired by RNA-Seq. These raw reads were filtered, and a total of 223.38 million clean reads (99.88%) were obtained from four sequencing libraries by using the Illumina HiSeq platform. The clean reads were aligned to the reference genome by using BWA software. The proportion of mapped reads to clean reads was 98.43%, 98.49%, 99.11% and 99.18% in LJ11, LJ25, L-pool and H-pool, respectively. Moreover, the sequencing depths were 10-fold, 10-fold, 67-fold and 80-fold in LJ11, LJ25, L-pool and H-pool, respectively. It indicated that the sequencing depth was higher in segregating pools than the parents, which corroborated the accuracy of the BSA analysis. The average of onefold coverage ratio was 96.06% (table 1). The 204 455 and 21 548 SNPs, including 3411 and 467 non-synonymous SNPs, were identified between LJ11 and LJ25, and the L-pool and H-pool, respectively. A total of 51 518 and 7766 indels were identified between LJ11 and LJ25, including 391 and 98 frameshift indels in the L-pool and H-pool, respectively (electronic supplementary material, tables S2 and S3).

**Table 1.** Overview of the BSA-Seq data.

sample	raw_read	adapter_per cent (%)	clean_reads	clean_base	mapped (%)	ave_depth	cov_ratio_1X (%)
LJ11	14 429 554	0.22	14 397 542	4 313 194 976	98.43	10	95.06
LJ25	14 128 180	0.19	14 100 600	4 224 137 016	98.49	10	95.54
L-pool	93 385 320	0.1	93 287 794	27 940 214 042	99.11	67	98.36
H-pool	102 166 895	0.09	102 063 410	30 571 374 716	99.18	80	95.29

### 2.4. Identification of the candidate regions related to the chilling tolerance at the booting stage

**Figure 4.**
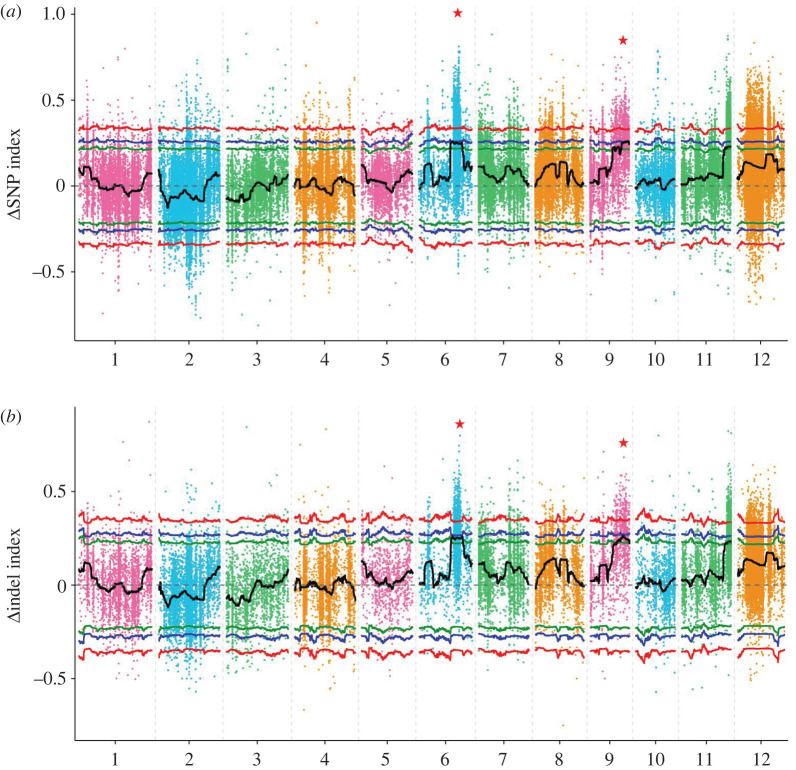
Manhattan plot analysis on the variation tendency of the ΔSNP index and Δindel index between H-pool and L-pool associated with SSRPPM distribution in the chromosomes: (*a*) ΔSNP index and (*b*) Δindel index. The red stars indicate the candidate association region related to SSRPPM. The number of *X*-axis represents the chromosome number. The red, blue and green curve lines represent the threshold of ΔSNP index and Δindel index.

The association analysis method of ΔSNP index and Δindel index was used to calculate the candidate regions of the genome related to the chilling tolerance at the booting stage. As depicted in the Manhattan plots (figure 4), ΔSNP-index method identified six candidate genomic regions that were significantly associated with the SSRPPM on chromosomes 6 and 9 between L-pool and H-pool. Its overall size was 3.67 Mb, and it included 509 annotated genes (electronic supplementary material, table S4). As per the Δindel-I index calculation method, 14 and 4 candidate genome regions were identified on chromosomes 6 and 9, respectively, which had a total size of 3.56 Mb and contained 421 annotated genes (electronic supplementary material, table S5). The candidate regions obtained from these two methods were intersected, and the final association regions were determined. It had a total size of 0.82 Mb and contained 99 genes, out of which 98 genes were annotated (electronic supplementary material, table S6). As shown in table 2, three associated regions were identified on chromosome 6, which had a size of 0.03 Mb (19.19–19.22 Mb), 0.01 Mb (19.33–19.34 Mb) and 0.4 Mb (19.38–19.78 Mb), and included 5, 2 and 28 genes, respectively. Another three regions were distributed on chromosome 9, which had a size of 0.08 Mb, 0.29 Mb and 0.01 Mb, and it included 15, 48 and 1 gene, respectively.

**Table 2.** Analysis of candidate regions associated with the SSRPPM.

chromosome_ID	start position	end position	size (Mb)	gene_number
6	19 190 000	19 220 000	0.03	5
6	19 330 000	19 340 000	0.01	2
6	19 380 000	19 780 000	0.4	28
9	19 640 000	19 720 000	0.08	15
9	19 850 000	20 140 000	0.29	48
9	20 170 000	20 180 000	0.01	1
total	—	—	—	99

### 2.5. Gene expression profile analysis and identification of candidate genes in the final association regions

**Figure 5.**
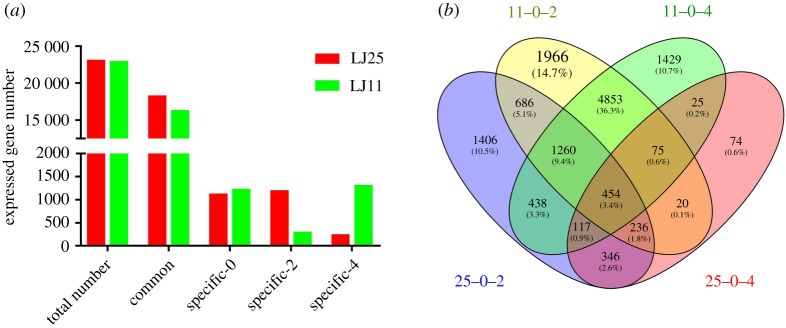
The overall gene expression pattern and DEGs identified in LJ25 and LJ11 under chilling stress: (*a*) expressed genes and (*b*) DEGs. ‘Total number’ represents the total genes detected in LJ25 and LJ11; ‘common’ represents the expressed genes commonly identified under all chilling stress periods in LJ25 and LJ11; ‘specific-0’, ‘specific-2’ and ‘specific-4’ represents the genes expressed explicitly on 0, 2 and 4 days of chilling stress periods in LJ25 and LJ11. ‘11-0-2’ and ‘11-0-4’ represent DEGs identified in LJ11 2 and 4 days post-chilling stress; ‘25-0-2’ and ‘25-0-4’ represent DEGs identified in LJ25, 2 and 4 days post-chilling stress.

A total of 156.02 Gb clean data, which included 1055.73 billion clean reads, were obtained from 18 samples through RNA-sequencing. The average GC content, Q20 and Q30 were 55.14%, 96.42% and 91.76%, respectively. Of the clean reads in samples, 79.55–84.3% were mapped to *Oryza sativa* L. ssp. *Japonica* genome by using TopHat2 software [44]. The low-expression genes (FPKM < 5) were filtered to obtain a total of 22 952 and 23 556 genes in LJ25 and LJ11, respectively. In LJ25, 18 419 genes were identified at the low-temperature treatment period, and 1100, 1174 and 217 genes were explicitly identified after days 0, 2 and 4 of the low-temperature treatment period, respectively. By contrast, in LJ11, 16 255 genes were identified throughout the treatment period, and 1124, 1039 and 1049 genes were identified explicitly after days 0, 2 and 4 of low-temperature treatment, respectively (figure 5*a*; electronic supplementary material, tables S7 and S8). In LJ11, a total of 9550 and 8651 DEGs were identified, 2 and 4 days post-chilling stress, respectively. By contrast, 4943 and 1347 DEGs were identified 2 and 4 days post-chilling stress, respectively, in LJ25 (figure 5*b*; electronic supplementary material, table S9).

**Figure 6.**
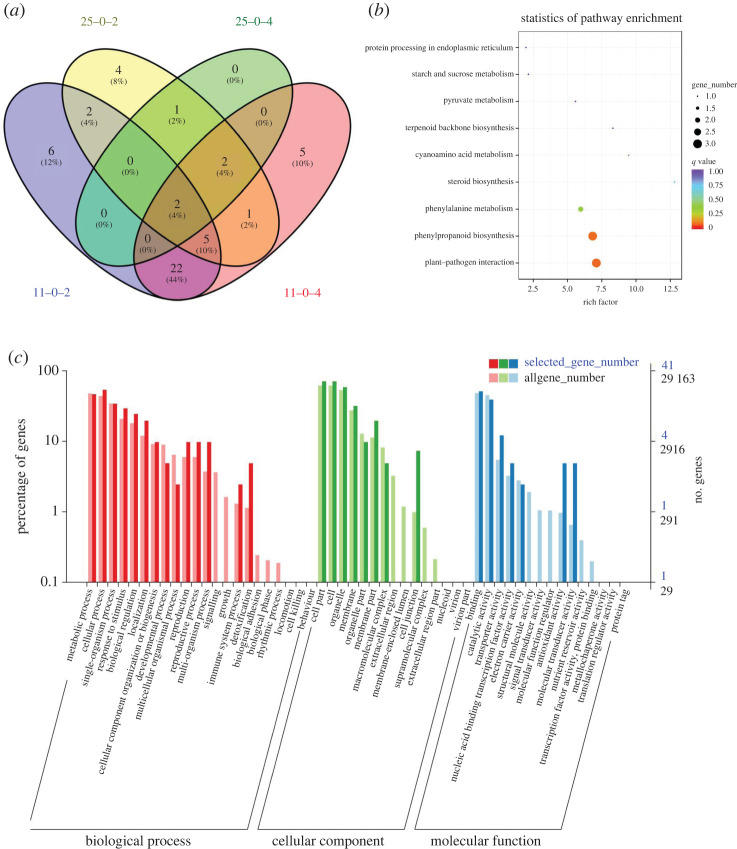
Analysis of DEGs identified in the candidate regions associated with chilling stress: (*a*) Venn-diagram-based analysis; (*b*) GO classification; (*c*) KEGG pathway enrichment analysis.

The outcomes of BSA-Seq and RNA-Seq analysis suggest that out of the 98 genes in the 0.82 Mb final association regions, 87 genes were expressed under chilling stress condition, of which 50 genes were differentially expressed (figure 6*a*; electronic supplementary material, table S10). Twenty-two DEGs (44% of the 50 DEGs) were identified in LJ11 throughout the chilling stress period, whereas only one DEG (2% of the 50 DEGs) was identified in LJ25. Os09g0507100 and Os09g0507600 encode squamosa promoter-binding-like protein and transmembrane emp24 domain-containing protein, were both differentially expressed in LJ11 and LJ25 throughout the chilling stress period.

GO classification analysis revealed that 50 DEGs belonged to GO categories of biological process, cellular component and molecular function. As shown in figure 6*c*, most of the DEGs were assigned to metabolic, cellular and single-organism processes of the biological process category. However, cell, cell parts and membrane in a cellular component and binding, catalytic and transporter activity were enriched in the molecular function category. KEGG pathway enrichment analysis was performed for 50 DEGs, and nine KEGG pathways were found to be significantly enriched (figure 6*b*), which contained a total of 10 DEGs (table 3). However, the majority of these DEGs were expressed in LJ11 only and not in LJ25 under chilling stress. *Os09g0511600*, also known as *Os9BGlu31*, encodes a glycoside hydrolase family GH1 transglycosidase. It was the only gene that was downregulated in LJ11 and upregulated in LJ25. Interestingly, besides the ‘phenylpropanoid biosynthesis' KEGG pathway, *Os09g0511600* was also enriched in ‘cyanoamino acid metabolism’ and ‘starch and sucrose metabolism’ pathway, which suggests the crucial involvement of *Os09g0511600* in response to chilling stress at the booting stage.

**Table 3.** Expression level of the DEGs in the nine KEGG pathway. ‘—’ indicates no significant expressed; ‘Inf’ indicates infinite.

#ID	11_0_2			11_0_4			25_0_2			25_0_4		
	FDR	log2FC	regulated	FDR	log2FC	regulated	FDR	log2FC	regulated	FDR	log2FC	regulated
*Os06g0527100*	0.55	0.19	normal	0.40	0.25	normal	9.69 × 10^−4^	1.09	up	0.56	0.34	normal
*Os09g0513600*	2.72 × 10^−18^	2.03	up	4.82 × 10^−9^	1.37	up	0.85	0.08	normal	8.00 × 10^−1^	0.17	normal
*Os09g0514200*	8.01 × 10^−7^	2.84	up	9.79 × 10^−10^	3.19	up	0.63	0.36	normal	0.15	1.04	normal
*Os09g0507550*	—	—	—	2.99 × 10^−13^	Inf	up	3.45 × 10^−4^	−3.63	down	7.66 × 10^−3^	−2.33	down
*Os09g0507500*	—	—	—	—	—	—	1.41 × 10^−6^	−3.82	down	8.84 × 10^−5^	−2.64	down
*Os09g0516500*	6.02 × 10^−5^	1.51	up	1.53 × 10^−5^	1.53	up	0.028617	−0.90	normal	0.25	−0.57	normal
*Os09g0514400*	1.33 × 10^−4^	−1.10	down	1.42 × 10^−8^	−1.63	down	0.24	−0.50	normal	0.21	−0.67	normal
*Os09g0516600*	3.34 × 10^−7^	−1.35	down	1.11 × 10^−5^	−1.15	down	8.28 × 10^−3^	0.82	normal	0.17	0.66	normal
*Os09g0511600*	2.87 × 10^−60^	−8.48	down	9.60 × 10^−6^	−1.31	down	8.62 × 10^−31^	3.62	up	0.15	3.35	normal
*Os09g0512700*	0.32	−0.41	normal	2.98 × 10^−3^	−1.14	down	0.02	−1.09	normal	0.35	−0.57	normal

### 2.6. qRT-PCR-based validation of DEGs

**Figure 7.**
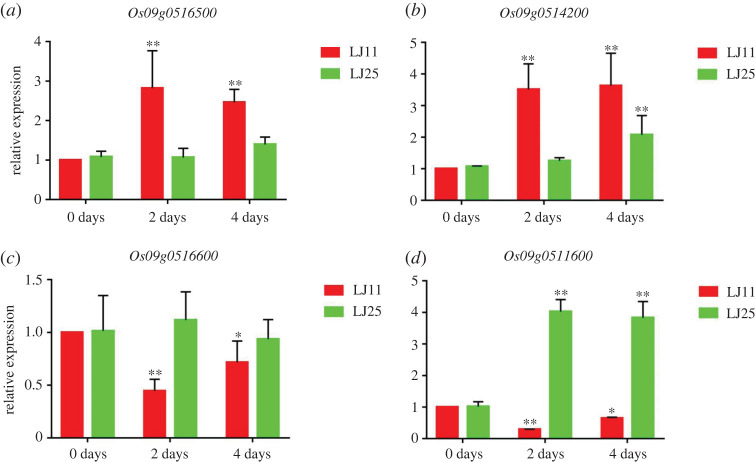
Quantitative real-time PCR-based validation of the relative expression levels of DEGs in the candidate regions associated with chilling stress. Values are the means (±s.d.) of three biological replicates. Asterisks represent statistically significant differences (*p < 0.05; **p < 0.01) between the control (0 days) and treatment groups (2 and 4 days) in LJ25 and LJ11, respectively.

The DEGs identified in the RNA-Seq study were selected for further validation. Four (*Os09g0514200*, *Os09g0516500*, *Os09g0516600* and *Os09g0511600*) out of the 10 DEGs enriched in KEGG pathways were selected for quantitative real-time PCR (qRT-PCR)-based validation. These DEGs showed a higher differential expression than the other six genes under chilling stress conditions at the booting stage. We found that the outcome of the qRT-PCR validation study was consistent with the RNA-Seq data, which indicates the reliability of the transcriptome analysis (figure 7).

## 3. Discussion

Rice (*Oryza sativa* L.) is more sensitive to chilling stress than other cereal crops. Due to its evolutionary pathway, rice production is hampered due to chilling stress in temperate areas [2], especially the ones with the low temperature at the critical booting stage [45]. Therefore, breeding of rice varieties that are tolerant to chilling conditions at the booting stage is an effective approach to improve the chilling tolerance and thus maintain high and stable yield of rice. In the current investigation, we chose a couple of *Japonica* rice cultivars with different chilling tolerance at the booting stage: LJ25 (chilling-tolerant) and LJ11 (chilling-sensitive). To construct an F_2_ segregating population and identify the QTLs or genes related to the chilling tolerance, both these varieties were cultivated in the Heilongjiang province of northeastern China. The chilling tolerance of rice at the booting stage is an overly complex trait [46]. Thus, its identification is comparatively difficult when compared with the other agronomic traits. The previous report has shown that the SSRs could be treated as an evaluation index of chilling damage of rice at the booting stage [9,43,47]. In this study, we calculated the SSRPP and SSRPPM to evaluate the chilling tolerance of the F_2_ population. Both SSRPP and SSRPPM exhibited similar trends in each individual plant under chilling stress conditions, which indicates that both of them could serve as effective evaluation indexes. However, the SSRPPM was selected to evaluate chilling damage in rice at the booting stage as it investigates the meiosis stage of tillers, and thus, it is more accurate than SSRPP.

Previous studies have reported that QTLs associated with chilling tolerance in rice could be present on different chromosomes, particularly, chromosomes 3, 4 and 7 [24,48–51]. Among these QTLs, *qLTB3* [9], *qCT8* [52] and *qCT7* [51] were fine mapped and *Ctb1*, *CTB4a* and *qPSR10* were cloned [23–25]. On chromosome 6, two QTLs, *qCTB6* [21] and *dth6* [53], which control spikelet fertility and heading time, were identified by SSR and restriction fragment length polymorphism (RFLP) markers on large intervals, but it was hard to use these two QTLs effectively for chilling-tolerant molecular breeding. Sun *et al*. [54] identified a new QTL (*qPSST6*) associated with chilling tolerance of rice at the booting stage within an interval of 1.81 Mb on chromosome 6 by using the BSA-Seq technology, which could be used in chilling tolerance breeding. SSR and RFLP markers that identified *qCTB9* [21] and *qPSST-9* [55] related to chilling tolerance at the booting stage were located at 5.1 cM and 10.8 cM interval, respectively, on chromosome 9. In the current study, three chilling-tolerant-associated regions with small intervals of 0.03, 0.01 and 0.4 Mb were identified on chromosome 6, containing 5, 2 and 28 genes, respectively, within the intervals. Another three regions were located on 0.08, 0.29 and 0.01 Mb interval on chromosome 9, containing 15, 48, and 1 gene, respectively. These regions were more elaborate than any other regions reported so far.

The conventional QTLs identification method is time-consuming. It requires a high expenditure of time as it involves fine mapping and map-based cloning of specific traits for the construction of advanced generation population and high-density linkage maps. The conventional method took more than 16 years for the completion of QTL mapping and gene cloning of C*tb1,* gene-related to chilling tolerance at the booting stage of rice [23,50,56,57]. When compared with the conventional method of QTLs identification, the BSA method remarkably reduces the population scale and identification cost by using the simplified procedure. Due to the accuracy and sensitivity of the sequencing, BSA-Seq serves as an efficient method to identify the minor genes with multiple sequencing depths. RNA-Seq is used to find novel genes and SNP loci, detection of gene expression levels and determination of fold change in DEG [58]. Gao *et al*. [59] mapped the purple-leaf-related gene *plr4* in the three BSA pools, which was found to be located on chromosome 4 within 27.9–31.1 Mb region. Subsequently, RNA-Seq was performed, which identified 12 DEGs in the interval where two genes were found to be downregulated, and they were selected as candidate genes [59]. In our study, an integrated BSA-Seq and RNA-Seq analysis successfully identified the candidate regions associated with the chilling tolerance of rice at the booting stage in 0.82 Mb interval, which contained 98 annotated genes and 50 DEGs from the F_2_ population. A total of 10 DEGs were identified as the candidate genes responsible for chilling stress. Therefore, this strategy provides an effective, potent and time-saving tool for QTL mapping and gene cloning, specifically for those QTLs or genes with minor effects on some traits and in the low-generation population.

In this study, we found that 10 DEGs were enriched in nine KEGG pathways. However, the majority of these genes were differentially regulated in LJ11 and normally expressed in LJ25. It indicates that chilling stress had a higher impact on LJ11 when compared with LJ25. Four DEGs showed either high expression levels or opposite expression patterns in LJ11 and LJ25. The DREB-CRT/DRE (dehydration-responsive element-binding proteins-C-repeat/dehydration-responsive elements) pathway is one of the most studied pathways involved in the chilling sensing and response in rice. This pathway is initiated through a Ca^2+^-influx signal. Firstly, Ca^2+^ is transduced inside the cells by calcium-dependent protein kinases (CDPKs), which activate the expression of downstream genes such as *OsMYB3R-2*, *OsMYB2* and *OsICE1/2*, initiating a chilling response [60]. *Os09g0514200* encodes a calcium-dependent protein kinase 32, which was upregulated in LJ11 while no differential expression was observed in LJ25. It suggests that it was involved in a chilling response. The *Os09g0514200* function in the chilling response has not been reported so far. Its cloning and functional validation might help with the chilling tolerance breeding. *Os09g0516600* encodes glyoxalase II, also known as *OsGLYII3*, was found to be overexpressed under multiple abiotic stress without chilling stress [61]. However, it was downregulated in LJ11 while normally expressed in LJ25, which suggests that this gene positively affects the chilling stress, and it could be used for chilling tolerance breeding. Starch serves as the energy reservoir for cells during the pollen germination, and its accumulation is crucial [62] during the early microspore stage. *Os09g0511600*, also known as *Os9BGlu31*(a), encodes a glycoside hydrolase family GH1 transglycosidase that transfers glucose in flavonoids, phytohormones and phenolic acids [63]. Moreover, it is overexpressed in senescent flag leaves, tender seeds and enhanced in rice seedlings in response to phytohormones treatment and drought stress. In this study, *Os09g0511600* was the only gene that was overly downregulated in LJ11 while significantly upregulated in LJ25 due to chilling stress. Besides, it was also enriched in the three KEGG pathways, which indicates that it may be a crucial gene in response to chilling stress of rice at the booting stage. However, further validation studies, including genetic transformation and phenotypic investigation, will facilitate the molecular breeding of rice for chilling tolerance.

## 4. Material and methods

### 4.1. Materials

In the current study, the F_2_ segregating population (245 individuals) was derived from an intraspecific cross between two *Japonica* rice varieties: ‘Longjing25’ (LJ25) and ‘Longjing11’ (LJ11). The regions associated with the chilling tolerance at the booting stage were identified in this population. LJ25 is a high chilling-tolerant variety, whereas LJ11 is a chilling-sensitive variety at the booting stage. Both of these *Japonica* rice varieties are the main cultivar in Heilongjiang province. As both varieties were in the tertiary temperate zone of Heilongjiang province, it ensured the same developmental stage of the F2-population offspring.

### 4.2. Methods

#### 4.2.1. Chilling tolerance evaluation

The F2 population and the parental lines for outdoors chilling tolerance evaluation were planted in the cool water irrigation pool of ‘Rice Research Institute of Heilongjiang Academy of Agricultural Sciences' in 2017. These were sown on 16 April and transplanted on 16 May. The transplantation size was 30 × 13.3 cm. The chilling stress treatment method was according to the previously described short-term low-temperature treatment method but with slight modifications [54]. The meiosis stage of pollen was recognized when the flag leaf's auricle was around 5 cm underneath the penultimate leaf's auricle. This stage of pollen is most sensitive to chilling stress during the booting stage [64]. The population was irrigated with cool water at this stage for chilling stress treatment. To ensure the accuracy of the treatment stage and the outcomes, tillers (select the main stem as far as possible) on each offspring of the population and the parents undergoing the meiosis were selected and marked. The population was irrigated with water (18°C) for 10 days (08.00 to 16.00 each day), and the water depth was maintained at 20–25 cm. The irrigation water was mixed with the water from the cool pool and warm pool. The probe and automatic sensing system measured the temperature of the water.

Chilling tolerance of parents for RNA-Seq was evaluated by the cool air treatment method [65]. Twenty plants of each cultivar were transplanted in plastic pots (25 cm in diameter, 23 cm in height); only the main culm of each plant was retained for synchronous growth, and each variety was planted in six pots. Before chilling treatment, LJ25 and LJ11 were grown in artificial climate chest, and the temperature was set to 28°C (day) and 22°C (night) with 12 h light/dark photoperiod and 80% relative humidity. The chilling treatment was as described previously by Satake & Hayase [64], where the temperature was set to 12°C. Moreover, half of the plants at this stage were transferred to another artificial climate chest at 12°C for 2 and 4 days and later returned to the original box until maturity. After maturity, the spikelet of each offspring in the pool was harvested to calculate the SSRPP and the SSRPPM based on the ratio of fertile grains to florets [9]. The variance was analysed using SigmaPlot software v. 12.5 (Systat Software Inc., San Jose, CA, USA). Duncan's multiple range test was employed for comparing the mean differences at *p* < 0.05.

#### 4.2.2. Genome DNA extraction, detection and segregating pools construction

From each offspring of the F2 generation of LJ25 and LJ11, 0.5 g leaf samples were collected. CTAB method was used for DNA extraction. The quality and quantity of the extracted DNA were verified by agarose gel electrophoresis and NanoDrop ND-1000 spectrophotometer, respectively. According to the SSRPPM results, each 50 plants with extremely high or low chilling tolerance were selected for the construction of two segregating pools, respectively. DNA samples of the 50 plants with extremely high or low chilling tolerance were mixed in an equal amount to generate the H-pool (high chilling tolerance) and L-pool (low chilling tolerance), respectively. The DNA samples of the parent lines were prepared as two pools for sequencing.

#### 4.2.3. BSA-Seq analysis

Libraries for the four DNA pools were prepared as per the Illumina's protocol for library preparation. The DNA samples were nicked into 350 bp length fragments randomly by ultrasonication, ligated with adapters, and purified. The DNA libraries were sequenced on an Illumina HiSeq™2000 platform (Beijing Biomarker Biotechnology Co., Beijing, China). The low-quality reads containing adaptors were filtered. The reads with more than 10% of missing bases and more than 50% of bases with Q-score lower than 10 were filtered, and the clean reads thus obtained were mapped to the Nipponbare reference genome (Oryza_sativa_IRGSP-1.0) using BWA software [66]. SAM tools and ANNOVAR software were used for SNP-calling and SNP annotation, respectively [66,67]. MarkDuplicates tool in Picard (http://sourceforge.net/projects/picard/) was used to remove the duplicate reads located on the reference genome. To detect SNP accurately, the local rearrangement and base mass value calibration was performed with the GATK software package [68], including small indels (1–5 bp). SnpEff software [69] was used for SNP annotation and determination of the effects of small indels on the genome (synonymous or non-synonymous mutations).

The SNP-index algorithm was used to identify the candidate regions of the genome associated with SSRPPM. It was also used to calculate the differences in allele frequency between bulked pools [35,70]. SNP index was the scale of short reads containing SNPs, different from the reference genome [71]. Δ(SNP index) was the SNP-index difference between H-pool and L-pool. Sliding window analysis was used to calculate the distribution of SNP index among the genome within 1 Mb width windows and 1 kb at each step. Δ(SNP index) was used to calculate the 1000 permutations in the genome, and candidate regions related to chilling tolerance were selected with 95% confidence [35]. Totally, all the analysis above was performed with the related tools on the online open platform BMKCloud (http://www.biocloud.com/).

#### 4.2.4. RNA-sequencing and analysis of gene expression profiles

Around 0.5 g of fresh young spikelets with 3.5–4.5 mm length were collected from the upper third of the panicles on days 0, 2 and 4 when the incubation temperature was set to 12°C. TRIzol total RNA extraction kit (Invitrogen, Carlsbad, CA, USA) was used for the total RNA extraction from fresh young spikelets as per the manufacturer's instructions. A total of 18 mixed RNA samples were extracted. These RNA samples were purified with the poly-T oligo (dT) beads. The mRNA was nicked into short fragments by using divalent cations under elevated temperature in NEBNext^®^ first-strand synthesis reaction buffer. These short mRNA fragments were used as the template. Random hexamer primer and MMuLV reverse transcriptase (RNase H) were used for the synthesis of the first strand, and DNA polymerase I and RNase H were used for the synthesis of the second strand of cDNA. Furthermore, the cDNA fragments were purified by using the AMPure XP system (Beckman Coulter, Beverly, WV, USA). The cDNA libraries were sequenced on the Illumina HiSeq™ 2500 platform with the paired-end technology (Beijing Biomarker Biotechnology Co., Beijing, China) to generate 150 bp paired-end reads.

Firstly, raw reads were generated in FASTQ format; adapters and low-quality reads were filtered to obtain the clean reads. These clean reads were later merged and mapped to the Nipponbare reference genome (Oryza_sativa_IRGSP-1.0) by using TopHat2 [44]. Fragments per kilobase of transcript per million mapped reads (FPKM) were estimated to quantify the gene expression levels [72]. DESeq R package v. 1.18.0 was used to perform differential expression analysis of the samples with the thresholds of fold change (FC) ≥ 3 and false discovery rate (FDR) ≤ 0.01. GOSeq R package was used to perform gene ontology (GO) analysis. The Kyoto Encyclopedia of Genes and Genomes (KEGG; http://www.genome.jp/kegg) is an open-access resource for understanding advanced functions and utilities of the biological system [73]. The KOBAS software was employed for the statistical KEGG pathway enrichment analysis of DEGs [74]. Also, all the analysis above was performed with the related tools on the online open platform BMKCloud (http://www.biocloud.com/).

#### 4.2.5. qRT-PCR-based validation of the expression profile of candidate genes

The expression levels of four genes (*Os09g0514200*, *Os09g0516500*, *Os09g0516600* and *Os09g0511600*) obtained by RNA-Seq in response to chilling stress were selected for the qRT-PCR-based validation. The primers were designed using Primer3 software. The list of primers is provided in electronic supplementary material, table S1. The qRT-PCR reactions were performed on an ABI 7500 qPCR thermal cycler (Applied Biosystems Inc., Carlsbad, CA, USA) using the QuantiNova™ SYBR^®^ Green PCR kit (Qiagen Inc., Duesseldorf, Germany), and each reaction was repeated thrice, both, biologically and technically. The qRT-PCR data were analysed using the 2^−ΔΔCT^ method [75] with the actin gene as the reference gene.

## 5. Conclusion

In this study, LJ25 (chilling-tolerant) and LJ11 (chilling-sensitive) were crossed to generate the F_2_ population, and later two pools with extremely high and low SSRPPM were constructed. BSA-Seq method identified a total of six candidate regions, which were associated with chilling tolerance, and these were distributed on chromosomes 6 and 9 at 0.82 Mb interval. This region contained 98 annotated genes. The RNA-Seq analysis of the parents led to the identification of 50 DEGs in the candidate regions. After the KEGG enrichment pathway analysis, 10 DEGs were found to be enriched in the nine significant KEGG pathways. Four genes between LJ25 and LJ11 showed either overexpression or different expression patterns, and these were selected as candidate genes in response to chilling stress, and further validated by qRT-PCR. A further in-depth analysis, including validation by gene cloning, genetic transformation and phenotypic investigations of these genes, could facilitate their use in chilling-tolerant molecular breeding.

## Supplementary Material

Table S1

Reviewer comments

## Supplementary Material

Table S2

## Supplementary Material

Table S3

## Supplementary Material

Table S4

## Supplementary Material

Table S5

## Supplementary Material

Table S6

## Supplementary Material

Table S7

## Supplementary Material

Table S8

## Supplementary Material

Table S9

## Supplementary Material

Table S10

## Supplementary Material

Table S11

## Data accessibility

All data generated or analysed during this study are included in this published article. Raw sequence data were deposited in the Sequence Read Archive (SRA) database (www.ncbi.nlm.nih.gov/sra), BioProject Accession: PRJNA495106 and PRJNA633525 (www.ncbi.nlm.nih.gov/bioproject).
