# Supplementary material for: Identification of candidate genes controlling chilling tolerance of rice in the cold region at the booting stage by BSA-Seq and RNA-Seq
Source: R Soc Open Sci. 2020 Nov 18;7(11):201081. doi: 10.1098/rsos.201081 (PMC7735347; doi:10.1098/rsos.201081)
Supplement: Reviewer comments [file rsos201081_review_history.pdf]

THE ROYAL SOCIETY
PUBLISHING

ROYAL SOCIETY
OPEN SCIENCE

Identification of candidate genes controlling chilling tolerance of rice in the cold region at the booting stage by BSA-Seq and RNA-Seq

Zhenhua Guo, Lijun Cai, Zhiqiang Chen, Ruiying Wang, Lanming Zhang, Shiwu Guan, Shuhua Zhang, Wendong Ma, Chuanxue Liu and Guojun Pan

Article citation details
R. Soc. open sci. **7**: 201081.
http://dx.doi.org/10.1098/rsos.201081

Review timeline

Original submission:	16 June 2020
Revised submission:	6 October 2020
Final acceptance:	14 October 2020

Note: Reports are unedited and appear as submitted by the referee. The review history appears in chronological order.

Review History

RSOS-201081.R0 (Original submission)

Review form: Reviewer 1

Is the manuscript scientifically sound in its present form?
Yes

Are the interpretations and conclusions justified by the results?
Yes

Is the language acceptable?
Yes

Do you have any ethical concerns with this paper?
No

Have you any concerns about statistical analyses in this paper?
No

Reports © 2020 The Reviewers; Decision Letters © 2020 The Reviewers and Editors; Responses © 2020 The Reviewers, Editors and Authors. Published by the Royal Society under the terms of the Creative Commons Attribution License http://creativecommons.org/licenses/by/4.0/, which permits unrestricted use, provided the original author and source are credited

Recommendation?
Accept with minor revision (please list in comments)

Comments to the Author(s)
In this study, Guo et al conducted BSA-Seq and RNA-Seq to identify candidate genes controlling chilling tolerance at booting stage in rice. This paper is well-written, however some issues need to be addressed for clarity, please see my comments below:
1. Supplementary Tables are off the number, Table S1 should be S2, S2 should be S3 and so on. Please double check and correct them.

2. Page 8, line 318 to 326: 0.5g spikelet sample were collected from each F2 offspring for DNA extraction and mix in equal amount to generate H-pool and L-pool. If I understand correctly, the same F2 offspring was also used to calculate the SSRPP and SSRPPM. Would the collection of 0.5g spikelet affect the SSRPP and SSRPPM? It's also not clear why using spikelet DNA but not leaf DNA for BSA-Seq?

Page 9, line 343: It was also used to calculate the differences in "genotype" frequency between bulked pools. Please check if using "allele" frequency is more appropriate in this case.

Review form: Reviewer 2

Is the manuscript scientifically sound in its present form?
Yes

Are the interpretations and conclusions justified by the results?
Yes

Is the language acceptable?
Yes

Do you have any ethical concerns with this paper?
Yes

Have you any concerns about statistical analyses in this paper?
No

Recommendation?
Accept with minor revision (please list in comments)

Comments to the Author(s)
The manuscript tired to identify the QTL related to cold tolerance in rice. The methodology combined with BSA-seq and RNA-seq is interesting, and the manuscript was well written, and logically.

Minor but major mistake.
Fig.2 x axis is wrong, please correct to the values of fertility%, not 1-18.

Minor
Need the population size F2 in M&M, how many line do you use?

Decision letter (RSOS-201081.R0)

We hope you are keeping well at this difficult and unusual time. We continue to value your support of the journal in these challenging circumstances. If Royal Society Open Science can assist you at all, please don't hesitate to let us know at the email address below.

Dear Dr Guo

On behalf of the Editors, we are pleased to inform you that your Manuscript RSOS-201081 "Identification of Candidate Genes Controlling Chilling Tolerance of Rice in the Cold Region at the Booting Stage by BSA-Seq and RNA-Seq" has been accepted for publication in Royal Society Open Science subject to minor revision in accordance with the referees' reports. Please find the referees' comments along with any feedback from the Editors below my signature.

We invite you to respond to the comments and revise your manuscript. Below the referees' and Editors' comments (where applicable) we provide additional requirements. Final acceptance of your manuscript is dependent on these requirements being met. We provide guidance below to help you prepare your revision.

Please submit your revised manuscript and required files (see below) no later than 7 days from today's (ie 30-Sep-2020) date. Note: the ScholarOne system will 'lock' if submission of the revision is attempted 7 or more days after the deadline. If you do not think you will be able to meet this deadline please contact the editorial office immediately.

Please note article processing charges apply to papers accepted for publication in Royal Society Open Science (https://royalsocietypublishing.org/rsos/charges). Charges will also apply to papers transferred to the journal from other Royal Society Publishing journals, as well as papers submitted as part of our collaboration with the Royal Society of Chemistry (https://royalsocietypublishing.org/rsos/chemistry). Fee waivers are available but must be requested when you submit your revision (https://royalsocietypublishing.org/rsos/waivers).

Thank you for submitting your manuscript to Royal Society Open Science and we look forward to receiving your revision. If you have any questions at all, please do not hesitate to get in touch.

Best regards,

Lianne Parkhouse
Editorial Coordinator
Royal Society Open Science
openscience@royalsociety.org

on behalf of Dr James Locke (Associate Editor) and Steve Brown (Subject Editor)
openscience@royalsociety.org

Associate Editor Comments to Author (Dr James Locke):

Thank you for the submission of your manuscript. It has now been evaluated by two referees, who are happy for publication following minor corrections. Please revise the manuscript according to their suggestions.

Reviewer comments to Author:
Reviewer: 1
Comments to the Author(s)

In this study, Guo et al conducted BSA-Seq and RNA-Seq to identify candidate genes controlling chilling tolerance at booting stage in rice. This paper is well-written, however some issues need to be addressed for clarity, please see my comments below:
1. Supplementary Tables are off the number, Table S1 should be S2, S2 should be S3 and so on. Please double check and correct them.

2. Page 8, line 318 to 326: 0.5g spikelet sample were collected from each F2 offspring for DNA extraction and mix in equal amount to generate H-pool and L-pool. If I understand correctly, the same F2 offspring was also used to calculate the SSRPP and SSRPPM. Would the collection of 0.5g spikelet affect the SSRPP and SSRPPM? It's also not clear why using spikelet DNA but not leaf DNA for BSA-Seq?

Page 9, line 343: It was also used to calculate the differences in "genotype" frequency between bulked pools. Please check if using "allele" frequency is more appropriate in this case.

Reviewer: 2
Comments to the Author(s)

The manuscript tired to identify the QTL related to cold tolerance in rice. The methodology combined with BSA-seq and RNA-seq is interesting, and the manuscript was well written, and logically.

Minor but major mistake.
Fig.2 x axis is wrong, please correct to the values of fertility%, not 1-18.

Minor
Need the population size F2 in M&M, how many line do you use?

===PREPARING YOUR MANUSCRIPT===

Your revised paper should include the changes requested by the referees and Editors of your manuscript. You should provide two versions of this manuscript and both versions must be provided in an editable format:
one version identifying all the changes that have been made (for instance, in coloured highlight, in bold text, or tracked changes);
a 'clean' version of the new manuscript that incorporates the changes made, but does not highlight them. This version will be used for typesetting.
Please ensure that any equations included in the paper are editable text and not embedded images.

Please ensure that you include an acknowledgements' section before your reference list/bibliography. This should acknowledge anyone who assisted with your work, but does not qualify as an author per the guidelines at https://royalsociety.org/journals/ethics-policies/openness/.

While not essential, it will speed up the preparation of your manuscript proof if you format your references/bibliography in Vancouver style (please see https://royalsociety.org/journals/authors/author-guidelines/#formatting). You should include DOIs for as many of the references as possible.

If you have been asked to revise the written English in your submission as a condition of publication, you must do so, and you are expected to provide evidence that you have received

language editing support. The journal would prefer that you use a professional language editing service and provide a certificate of editing, but a signed letter from a colleague who is a native speaker of English is acceptable. Note the journal has arranged a number of discounts for authors using professional language editing services (https://royalsociety.org/journals/authors/benefits/language-editing/).

===PREPARING YOUR REVISION IN SCHOLARONE===

To revise your manuscript, log into https://mc.manuscriptcentral.com/rsos and enter your Author Centre - this may be accessed by clicking on "Author" in the dark toolbar at the top of the page (just below the journal name). You will find your manuscript listed under "Manuscripts with Decisions". Under "Actions", click on "Create a Revision".

Attach your point-by-point response to referees and Editors at Step 1 'View and respond to decision letter'. This document should be uploaded in an editable file type (.doc or .docx are preferred). This is essential.

Please ensure that you include a summary of your paper at Step 2 'Type, Title, & Abstract'. This should be no more than 100 words to explain to a non-scientific audience the key findings of your research. This will be included in a weekly highlights email circulated by the Royal Society press office to national UK, international, and scientific news outlets to promote your work.

At Step 3 'File upload' you should include the following files:
-- Your revised manuscript in editable file format (.doc, .docx, or .tex preferred). You should upload two versions:
1) One version identifying all the changes that have been made (for instance, in coloured highlight, in bold text, or tracked changes);
2) A 'clean' version of the new manuscript that incorporates the changes made, but does not highlight them.
-- An individual file of each figure (EPS or print-quality PDF preferred [either format should be produced directly from original creation package], or original software format).
-- An editable file of each table (.doc, .docx, .xls, .xlsx, or .csv).
-- An editable file of all figure and table captions.
Note: you may upload the figure, table, and caption files in a single Zip folder.
-- Any electronic supplementary material (ESM).
-- If you are requesting a discretionary waiver for the article processing charge, the waiver form must be included at this step.
-- If you are providing image files for potential cover images, please upload these at this step, and inform the editorial office you have done so. You must hold the copyright to any image provided.
-- A copy of your point-by-point response to referees and Editors. This will expedite the preparation of your proof.

At Step 6 'Details & comments', you should review and respond to the queries on the electronic submission form. In particular, we would ask that you do the following:
-- Ensure that your data access statement meets the requirements at https://royalsociety.org/journals/authors/author-guidelines/#data. You should ensure that you cite the dataset in your reference list. If you have deposited data etc in the Dryad repository, please only include the 'For publication' link at this stage. You should remove the 'For review' link.
-- If you are requesting an article processing charge waiver, you must select the relevant waiver option (if requesting a discretionary waiver, the form should have been uploaded at Step 3 'File upload' above).
-- If you have uploaded ESM files, please ensure you follow the guidance at https://royalsociety.org/journals/authors/author-guidelines/#supplementary-material to include a suitable title and informative caption. An example of appropriate titling and captioning may be found at https://figshare.com/articles/Table_S2_from_Is_there_a_trade-

off_between_peak_performance_and_performance_breadth_across_temperatures_for_aerobic_scope_in_teleost_fishes_/3843624.

At Step 7 'Review & submit', you must view the PDF proof of the manuscript before you will be able to submit the revision. Note: if any parts of the electronic submission form have not been completed, these will be noted by red message boxes.

Author's Response to Decision Letter for (RSOS-201081.R0)

See Appendix A.

Decision letter (RSOS-201081.R1)

We hope you are keeping well at this difficult and unusual time. We continue to value your support of the journal in these challenging circumstances. If Royal Society Open Science can assist you at all, please don't hesitate to let us know at the email address below.

Dear Dr Guo,

It is a pleasure to accept your manuscript entitled "Identification of Candidate Genes Controlling Chilling Tolerance of Rice in the Cold Region at the Booting Stage by BSA-Seq and RNA-Seq" in its current form for publication in Royal Society Open Science. The comments of the reviewer(s) who reviewed your manuscript are included at the foot of this letter.

Please ensure that you send to the editorial office an editable version of your accepted manuscript, and individual files for each figure and table included in your manuscript. You can send these in a zip folder if more convenient. Failure to provide these files may delay the processing of your proof. You may disregard this request if you have already provided these files to the editorial office.

You can expect to receive a proof of your article in the near future. Please contact the editorial office (openscience_proofs@royalsociety.org) and the production office (openscience@royalsociety.org) to let us know if you are likely to be away from e-mail contact -- if you are going to be away, please nominate a co-author (if available) to manage the proofing process, and ensure they are copied into your email to the journal.

Due to rapid publication and an extremely tight schedule, if comments are not received, your paper may experience a delay in publication.

Please see the Royal Society Publishing guidance on how you may share your accepted author manuscript at https://royalsociety.org/journals/ethics-policies/media-embargo/.

Thank you for your fine contribution. On behalf of the Editors of Royal Society Open Science, we look forward to your continued contributions to the Journal.

Kind regards,
Anita Kristiansen
Editorial Coordinator

Royal Society Open Science
openscience@royalsociety.org

on behalf of Dr James Locke (Associate Editor) and Steve Brown (Subject Editor)
openscience@royalsociety.org

Associate Editor Comments to Author (Dr James Locke):
Comments to the Author:
Thank you for your revisions - the paper appears ready for publication. One minor question - the figure 2 axis refers to 'Seeding setting rates' and you refer to this as SSRPP in the legend. Should it be 'Seed setting rates', as this is how you define SSRPP in the paper. If so please correct 'seeding' to 'seed' in proofs for the figure axis and legend.

Follow Royal Society Publishing on Twitter: @RSocPublishing
Follow Royal Society Publishing on Facebook:
https://www.facebook.com/RoyalSocietyPublishing.FanPage/
Read Royal Society Publishing's blog:
https://royalsociety.org/blog/blogsearchpage/?category=Publishing

Appendix A

Dear editor:

Thank you for arranging a timely review of our manuscript (RSOS-201081.R1), entitled *Identification of Candidate Genes Controlling Chilling Tolerance of Rice in the Cold Region at the Booting Stage by BSA-Seq and RNA-Seq*. We are pleased to know that our study received highly favorable comments. We have carefully evaluated the reviewers' comments and thoughtful suggestions, responded to these suggestions point-by-point, and revised the manuscript accordingly. Here, we attached a revised manuscript in Word format with tracked changes for your approval.

We sincerely hope our revised manuscript meets the standard of Royal Society Open Science and will be accepted for publication.

Yours sincerely

Zhenhua Guo

Associate Editor Comments to Author (Dr James Locke):

Thank you for the submission of your manuscript. It has now been evaluated by two referees, who are happy for publication following minor corrections. Please revise the manuscript according to their suggestions.

Response: Thanks for editor' s constructive suggestions on this manuscript. We have revised the manuscript according to the suggestions of referees as follows.

Comments to Author:

Reviewer comments to Author:

Reviewer: 1

Comments to the Author(s)

In this study, Guo et al conducted BSA-Seq and RNA-Seq to identify candidate genes controlling

chilling tolerance at booting stage in rice. This paper is well-written, however some issues need to be addressed for clarity, please see my comments below:

1. Supplementary Tables are off the number, Table S1 should be S2, S2 should be S3 and so on. Please double check and correct them.

Response: Thanks for reviewer' s kindly reminding. We have checked the Supplementary Tables and corrected them in our revised manuscript.

2. Page 8, line 318 to 326: 0.5g spikelet sample were collected from each F2 offspring for DNA extraction and mix in equal amount to generate H-pool and L-pool. If I understand correctly, the same F2 offspring was also used to calculate the SSRPP and SSRPPM. Would the collection of 0.5g spikelet affect the SSRPP and SSRPPM? It's also not clear why using spikelet DNA but not leaf DNA for BSA-Seq?

Response: Thanks very much for reviewer' s rigorous and meticulous comment. I'm so sorry that it's a clerical error on the description of the sample collected from each F2 offspring for DNA extraction. Actually, we collected the leaf of each sample from each F2 offspring for DNA extraction and mix in equal amount to generate H-pool and L-pool. The spikelet of each F2 offspring was used to calculate the SSRPP and SSRPPM. I apologize for this inaccurate statement and I have changed it in our revised manuscript.

Page 9, line 343: It was also used to calculate the differences in "genotype" frequency between bulked pools. Please check if using "allele" frequency is more appropriate in this case

Response: Thank you very much for this comment. According to your advice, we have changed the "genotype" frequency to "allele" frequency in our revised manuscript.

Reviewer: 2

Comments to the Author(s)

The manuscript tired to identify the QTL related to cold tolerance in rice. The methodology combined with BSA-seq and RNA-seq is interesting, and the manuscript was well written, and logically.

Minor but major mistake.

Fig.2 x axis is wrong, please correct to the values of fertility%, not 1-18.

Response: Thanks very much for reviewer's comment. According to your advice, we have corrected the x axis of Fig.2 in our revised manuscript.

Minor

Need the population size F2 in M&M, how many line do you use?

Response: Thanks very much for reviewer's comment. In this research, a total of 245 F2 plants were derived from a cross between LJ25 and LJ11, and we have added this in the Materials and Methods in our revised manuscript.
